# Local adaptation constrains the distribution potential of heat-tolerant *Symbiodinium* from the Persian/Arabian Gulf

**DOI:** 10.1038/ismej.2015.80

**Published:** 2015-05-19

**Authors:** Cecilia D'Angelo, Benjamin C C Hume, John Burt, Edward G Smith, Eric P Achterberg, Jörg Wiedenmann

**Affiliations:** 1 Coral Reef Laboratory, Ocean and Earth Science, University of Southampton, Waterfront Campus, National Oceanography Centre, Southampton, UK; 2 Marine Biology Laboratory, Centre for Genomics and Systems Biology, New York University Abu Dhabi, Abu Dhabi, UAE; 3 GEOMAR, Helmholtz Centre for Ocean Research, Kiel, Germany; 4 Institute for Life Sciences, University of Southampton, Highfield Campus, Southampton, UK

## Abstract

The symbiotic association of corals and unicellular algae of the genus *Symbiodinium* in the southern Persian/Arabian Gulf (PAG) display an exceptional heat tolerance, enduring summer peak temperatures of up to 36 °C. As yet, it is not clear whether this resilience is related to the presence of specific symbiont types that are exclusively found in this region. Therefore, we used molecular markers to identify the symbiotic algae of three *Porites* species along >1000 km of coastline in the PAG and the Gulf of Oman and found that a recently described species, *Symbiodinium thermophilum*, is integral to coral survival in the southern PAG, the world's hottest sea. Despite the geographic isolation of the PAG, we discovered that representatives of the *S. thermophilum* group can also be found in the adjacent Gulf of Oman providing a potential source of thermotolerant symbionts that might facilitate the adaptation of Indian Ocean populations to the higher water temperatures expected for the future. However, corals from the PAG associated with *S. thermophilum* show strong local adaptation not only to high temperatures but also to the exceptionally high salinity of their habitat. We show that their superior heat tolerance can be lost when these corals are exposed to reduced salinity levels common for oceanic environments elsewhere. Consequently, the salinity prevailing in most reefs outside the PAG might represent a distribution barrier for extreme temperature-tolerant coral/*Symbiodinium* associations from the PAG.

## Introduction

Tropical coral reefs represent a major natural resource that depends on the habitat-forming scleractinian coral species and their symbiosis with dinoflagellate algae of the genus *Symbiodinium* (zooxanthellae). Damage to the algal symbiont can result in the breakdown of this association leading to often fatal coral bleaching. Episodes of mass bleaching events that have contributed to global reef degradation have been linked to high-temperature anomalies. The threshold temperature for heat-induced bleaching typically lies only 1 °C above the average maximum summer temperature for a given region (Goreau and Hayes, 1994; Baker *et al.*, 2008) and will be reached more frequently as sea surface temperatures rise. The resulting increased frequency of mass bleaching has been predicted to devastate coral reef populations on a global scale within decades (Baker *et al.*, 2008; van Hooidonk *et al.*, 2013; Logan *et al.*, 2014).

Furthermore, environmental factors such as reduced salinity and changes in the concentrations of dissolved inorganic nutrients can promote bleaching (Goreau, 1964; Coles, 1992; Wiedenmann *et al.*, 2013; D'Angelo and Wiedenmann, 2014). Coral reef survival will strongly depend on the adaptive and acclimatory responses of corals and their symbionts to the pressure of global warming (Logan *et al.*, 2014). The understanding of these adaptive processes requires the knowledge of the distribution of species with a high-temperature stress tolerance.

The highest known bleaching thresholds are found in coral communities in the Persian/Arabian Gulf (PAG), which represent a biogeographic subset of Indo-Pacific coral communities (Coles, 2003; Sheppard *et al.*, 1992). Despite their exceptional capacity to survive higher temperature as compared to corals from elsewhere, PAG corals remain susceptible to bleaching when temperatures exceed their local thresholds. Recently, major bleaching events have resulted in a strong decline of PAG coral communities (Burt *et al.*, 2013; Coles and Riegl, 2013; Riegl *et al.*, 2011; Sheppard and Loughland, 2002). Nevertheless, while conspecifics or close relatives of PAG corals usually bleach and die at temperatures ⩾32 °C elsewhere (Coles and Riegl, 2013), coral symbioses in the PAG endure summer peak temperatures of up to 36 °C (Coles and Riegl, 2013; Hume *et al.*, 2013). Notably, they also need to withstand unusually low temperatures which can drop below 20 °C during winter (Coles, 1992; Coles, 2003; Sheppard *et al.*, 1992).

*Symbiodinium* clade D, certain members of which are considered to increase the heat tolerance of its coral hosts, has been found in the northwestern (off Saudi Arabia) and northeastern (off Iran) PAG (Baker *et al.*, 2004; Ghavam Mostafavi *et al.*, 2007; LaJeunesse *et al.*, 2014), prompting the hypothesis that this symbiont might be responsible for the heat tolerance in this region. We found, however, that *Symbiodinium* of the internal transcribed spacer 2 (ITS2)-type (‘subclade') C3 is the year-round prevalent symbiont in corals of Saadiyat reef in the southern PAG where clade D is essentially absent (Hume *et al.*, 2013; Hume *et al.*, 2015). This was surprising, since members of subclade C3 are generally considered to be ‘cosmopolitan, thermally sensitive generalists' (Lajeunesse, 2005). Further investigation revealed that the representatives of this ‘subclade' from the PAG are in fact a separate species, *Symbiodinium thermophilum* (Hume *et al.*, 2015). The genetically distinct nature of this species became obvious from the analysis of the non-coding region of the chloroplast *psbA* gene (*psbA*^ncr^) and was confirmed by the specific sequences of domain V of the chloroplast large subunit ribosomal DNA (cp23S) and the mitochondrial cytochrome b gene (*cob*; Hume *et al.*, 2015). Furthermore, we have described an ITS2 variant (termed C3*-Gulf ITS2 variant*), which contains a characteristic 8-bp sequence duplication and occurs at low abundance among *S. thermophilum* populations (Hume *et al.*, 2015). These results are in agreement with previous findings, which demonstrate that subclade C3 consists of different species lineages that cannot be resolved by molecular phylogenetic analysis using solely the ITS2 region of the nuclear ribosomal DNA sequences (LaJeunesse and Thornhill, 2011; Thornhill *et al.*, 2014).

The ability to distinguish *S. thermophilum* from other subclade C3 symbionts enabled us to assess for the first time whether this heat-tolerant symbiont is widely distributed in the southern PAG and can also occur outside this waterbody. This is of particular interest given the geographic isolation of the PAG from the adjacent Gulf of Oman and the wider Indian Ocean through the only 42-km-wide Strait of Hormuz. Because of the restricted water exchange, shallow depths (mean 30 m), high evaporation rates and limited freshwater input, the PAG waters are characterised by high salinities (often 42–44). The resulting halocline circulation is sustained by a net inflow of surface water into the PAG and limited outflow of heavier, more saline bottom waters (Sheppard *et al.*, 1992; Johns *et al.*, 1999; Yao and Johns, 2010). Consequently, PAG corals and their algal symbionts have to cope with the highest salinity levels reported globally for coral reef ecosystems (Coles, 2003; Feary *et al.*, 2013). Furthermore, the net inward flow of seawater likely inhibits the movement of coral larvae and other pelagic organisms from the PAG into adjacent seas (Sheppard *et al.*, 1992).

To assess whether *S. thermophilum* is widely distributed in the southern PAG and whether it can also occur outside the PAG, we focused our study on *Porites lobata*, *P. lutea* and *P. harrisoni* since all individuals of these corals analysed so far in the Southern PAG hosted this symbiont species as the year-round dominant partner (Hume *et al.*, 2015). Moreover, the genus *Porites* represents an important framework builder in most reefs of the world including the PAG (Burt *et al.*, 2008; Loya *et al.*, 2001) and *P. lobata* hosting *S. thermophilum* is a well-characterised laboratory model for physiological experiments with PAG corals (Hume *et al.*, 2013).

## Materials and methods

### Sample collection and processing

Samples of *P. lobata*, *P. lutea* and *P. harrisoni* colonies were collected at different sites within the PAG, the Strait of Hormuz and the Gulf of Oman in September 2012 and March 2013. Samples were removed from the top of the colonies from a depth range of 2–7 m and transferred to the surface in individual containers for immediate fixation in ethanol absolute or freezing in dry ice. For best results, genomic DNA was extracted using a previously described cetyltrimethylammonium bromide-based protocol (Hume *et al.*, 2013). DNA was dissolved in deionized water and stored frozen at −20 °C. For molecular taxonomy purposes, the ITS1-5.8 S-ITS2 region of the rDNA operon of zooxanthellae was amplified using the primers SYM-VAR_FWD and SYM_VAR_REV and PCR conditions as already published (Hume *et al.*, 2013). These primers were previously optimised to avoid contaminations of the amplicon with *Porites* host sequences. The PCR products were purified with the Jetquick Gel Extraction Spin Kit (GenoMed, Leesburg, FL, USA) and stored frozen for downstream analyses.

### Determination of zooxanthellae phylotypes

To determine the phylotypes of zooxanthellae by sequencing, the PCR-amplified ITS1-5.8 S-ITS2 region of the rDNA operon was cloned using the StrataClone PCR cloning kit (Agilent, Santa Barbara, CA, USA) and plasmid DNA was prepared from *Escherichia coli* colonies using a Fermentas GeneJET plasmid Miniprep kit (Thermo Scientific, Waltham, MA, USA). Sequencing services were provided by Macrogen Inc (Seoul, South Korea) and Eurofins Genomics (Ebersberg, Germany) and the 220-bp sequence of the ITS2 region was subjected to BLAST searches (www.ncbi.nlm.nih.gov/blast). Subsequently, the ITS2 phylotype of the identical/most similar reference sequences available in GenBank was assigned to the query sequence. Representative ITS2 sequences including those of (sub)clades C3, C3*-Gulf ITS2 variant*, D and C15 cluster from every sampled region were submitted to GenBank (accession numbers: KJ563083-KJ563104). Clade information for *Montipora foliosa* was retrieved from Wiedenmann *et al.* (2013). The 55 coral colonies examined in this study returned 519 zooxanthellae ITS2 sequences. Sequences of 25 colonies from Dalma, Saadiyat and Umm Al Quwain previously used in Hume *et al.* (2015) to define *S. thermophilum* were included for comparison.

To determine the dominant zooxanthellae (sub)clade present in *Porites* colonies, the ITS2 region was analysed by PCR and denaturing gradient gel electrophoresis (DGGE) as detailed in Supplementary Methods. Briefly, the ITS2 region was amplified by using genomic DNA as template in a PCR reaction with the primer pair SYM_VAR_5.8SII and SYM_VAR_Clamp. DGGE analysis was conducted using a Bio-Rad DCode system (Bio-RAD, Hercules, CA, USA) for DGGE with a model 475 gradient former. The taxonomic information contained in characteristic gel patterns was established by subjecting DNA extracted from representative bands to further analyses including sequencing. Thereby, DNA markers of all *Symbiodinium* (sub)clades relevant to this study were generated and subsequently processed along with the samples. Two representative DGGE gels are shown in Supplementary Figure S1. The DGGE phylotypes were determined for 305 coral colonies. The dominant phylotypes of 22 colonies from Dalma, Saadiyat and Umm Al Quwain from Hume *et al.* (2015) were included for comparison.

### *psbA*^ncr^ amplification and molecular phylogeny

The non-coding region of the chloroplast *psbA* gene (*psbA*^ncr^) was amplified from zooxanthellae of 109 *P. lobata*, *P. lutea* and *P. harrisoni* coral colonies collected at sites along ~1000 km of UAE and Oman coastline from September 2012 to March 2013. Out of 109 coral colonies analysed for this study, 22 were previously phylotyped (Hume *et al.*, 2015) and included for comparison.

The *psbA*^ncr^ was amplified from frozen DNA using the primers psbAFor_1 and psbARev_1 with thermal cycling as described in LaJeunesse and Thornhill (2011). PCR reaction conditions were as described for the SYM_VAR_FWD and SYM_VAR_REV primer pair in Hume *et al.* (2013).

Amplicons were directly sequenced using the internal primer psbA_int_Fwd, 5′-CTAGGTATGGAAGTGATGCATG-3' sequencing services were provided by Eurofins Genomics. Sequence chromatograms were checked by eye for potential sequencing errors. Any sequence with a chromatogram characteristic of multiple PCR products (for example, where several peaks were registered for a single nucleotide position or where reading frame shifts were apparent) was discarded from further analysis (~7.5% of all directly sequenced PCR amplicons).

The phylogenetic resolution of *psbA*^ncr^ sequences of ITS2-type C3 zooxanthellae associated with *Porites* spp. from the PAG and the Gulf of Oman (127 sequences) was assessed in relation to *psbA*^ncr^ sequences of ITS2-type C3 zooxanthellae and closely related variants from corals elsewhere retrieved from databases (266 sequences). Sequences were first aligned using ClustalW in MEGA6 (Molecular Evolutionary Genetics Analysis software, http://www.megasoftware.net/mega.php), checked by eye and edited manually. Phylogenies were estimated through Bayesian inference using MrBayes 3.2.24 (MrBayes:Bayesian Inference of Phylogeny software, http://mrbayes.sourceforge.net/index.php) applying the Jukes-Cantor model with a gamma-shaped distribution with invariable sites. The MCMC (Markov Chain Monte Carlo) analyses were run for 7.0 × 10^6^ generations, sampling every 500 generations. A relative burn-in of 0.25 was used in calculating a 50% majority rule consensus tree. Nodal support was estimated using posterior probabilities (PPs). A non-Gulf C3-type *psbA*^ncr^ sequence collected in the Great Barrier Reef was used as an outgroup (accession number JQ043643). Pairwise genetic distances between *psbA*^ncr^ haplotypes were calculated with MEGA6 using all substitutions and with gaps considered via pairwise deletion.

### Coral culture and physiological experiments

Replicate colonies (Ø ~3.5 cm) of two previously characterised laboratory strains of PAG *P. lobata* originating from Saadiyat reef, UAE (Hume *et al.*, 2013) associated with *S. thermophilum* and of Indo-Pacific representatives of *M. foliosa*, *Montipora* sp. and *P. lichen* were co-cultured in compartments of our experimental mesocosm at the National Oceanography Centre, Southampton at a constant temperature of 27.5 °C and a 12 h/12 h light/dark cycle at salinity levels of 42 and 36.5 (Practical Salinity Scale 1978, PSS-78). The key element of this approach is that the replicate colonies of the PAG and Indo-Pacific species were not assessed in isolation, but kept/treated always in parallel in the same experimental compartments. In this setting, the corals exposed to their native salinity did not only serve as controls for the salinity treatment, but also at the same time as ‘environmental controls' for the overall conditions of the experimental system.

Both compartments were fully equipped to sustain stable coral growth (D'Angelo and Wiedenmann, 2012; Hume *et al.*, 2013). To keep the environmental conditions comparable, three times per week, 30% of the water was exchanged between the two systems. During the procedure, water from the high-salinity system (~0.87 litre per litre of the intended exchange volume) was topped up with reverse osmosis water (~0.13 litre per litre of the intended exchange volume) to reach the required salinity of 36.5. The water from the reduced salinity system could be directly incorporated into the high-salinity system, also to compensate for evaporation losses. The regular mixing of the two waterbodies kept water parameters other than salinity comparable.

When corals were to be studied at a salinity level different from that of their origin, they were first acclimated for 4 weeks to the altered salinity. To analyse heat-stress responses, the temperature of flow-through compartments integrated in the high and reduced salinity systems were ramped up by 0.5 °C per day and kept constant at 32 °C. The heat treatment tanks constantly circulated water from the control tanks, which was heated up when entering the experimental tank, thereby exposing the corals to the same water chemistry as in the respective controls. The physiological performance of the corals was assessed in two independent 5-month experiments, one measuring the colony weight, the other the live tissue cover, the heat-stress treatment were carried out in parallel. Growth or decay of the corals was quantified either by determining the weight after a 30-s drip-off period on absorbent tissue or by calculating the area of the corals covered with live tissue using ImageJ (http://rsbweb.nih.gov/ij/) analysis of digital photographs. Micrographs were produced with a Leica MZ10 stereo microscope (Leica Microsystems, Wetzlar, Germany).

### Data analyses

The different strains/species of model corals were each represented by five replicate colonies in the treatments and in the controls of experiments carried out at ambient temperature. Four replicate colonies of each strain/species were used in the treatment and control of the heat-stress experiments to keep the number of killed animals small. Data of coral growth determined by changes in weight and live tissue cover were normalised to the initial values recorded after the acclimation period. The normalised data were fitted to theoretical models as defined in Supplementary Table S1. Two-sample *t*-tests were performed to evaluate the significance of the difference between the values obtained in each experiment at different salinity treatments. All analyses were performed using Origin 8.6.0 software (OriginLab Corporation, Northampton, MA, USA).

## Results

### Biogeographic distribution of extreme temperature-tolerant coral–zooxanthellae associations

**Figure 1:**
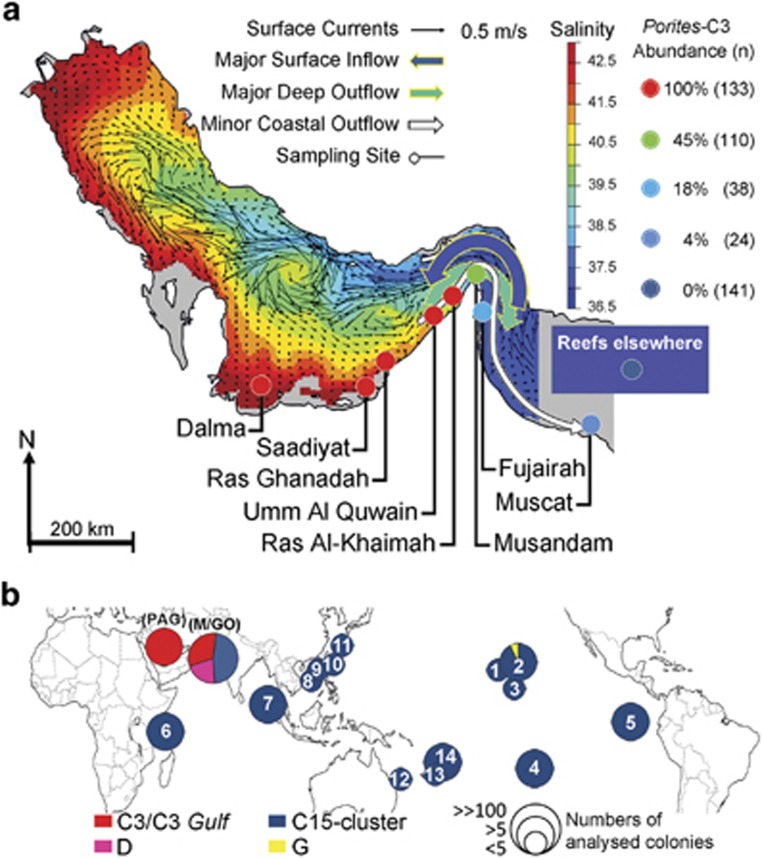
Global distribution of *Porites* spp.–*Symbiodinium* associations. (**a**) Schematic depiction of the sampling sites and oceanographic conditions in the PAG and the western Gulf of Oman. Seasonally averaged (July to September) surface salinity fields (coloured contour plot) and currents (black arrows) are overlaid using the 10-year model output (climatological run) from Yao and Johns (2010). The major surface inflow and deep outflow currents that connect the PAG with the Gulf of Oman are denoted by the thick arrows coloured according to their prevailing salinity. A minor coastal surface outflow (Johns *et al.*, 1999) is represented by a solid white arrow. The sampling sites of the present study in the southern PAG, Musandam and the Gulf of Oman are highlighted by coloured filled circles. The percentage of *Porites* spp. (*P. lobata*, *P. lutea* and *P. harrisoni*) colonies that harbour C3 (determined by DGGE; Supplementary Table S2) in the different regions is colour coded in the figure and defined in the legend. The total number of analysed colonies is indicated in brackets (*n*), sample sizes of the individual locations are detailed in Supplementary Figure S2. Literature data for reefs elsewhere are specified in Supplementary Table S3. Salinity and current imagery was kindly provided by W.E. Johns, University of Miami. (**b**) Global distribution of *P. lobata/P. lutea* associations with *Symbiodinium* cladal/subcladal types (as defined by the internal transcribed spacer 2 (ITS2) region of the *Symbiodinium* rDNA operon). Pie chart size and filled colours represent the number of individual coral colony associations identified and ITS2 cladal/subcladal type, respectively. Integers within the pie charts refer to the sampling regions (Supplementary Table S3). Data for the PAG (G) and Musandam/Gulf of Oman (M/GO) from the present study are included for comparison (Supplementary Table S2).

The answer to the question whether the heat tolerance of PAG corals is related to the presence of specific symbiont types is an integral part of the understanding of the exceptional resilience of corals in this region. Therefore, we identified the dominant dinoflagellate symbionts by analysing the rDNA ITS2 region (Supplementary Methods) of three *Porites* species (*P. lobata*, *P. lutea* and *P. harrisoni*) sampled along >1000 km of coastline of the southern PAG and the Gulf of Oman (Figure 1). In the PAG (highest temperatures and high-salinity water), 100% of these corals (>130 independent colonies from five locations) host *Symbiodinium* C3-type zooxanthellae (Figure 1a; Supplementary Figure S2 and Supplementary Table S2). Our analysis of a large set of symbiont data revealed that this specific partnership has not been found in coral reefs elsewhere in the world where *P. lobata/P. lutea* host almost exclusively C15-type *Symbiodinium* (217 analysed colonies, Figure 1b; Supplementary Figure S2 and Supplementary Table S3). We also detected representatives of this unusual C3-type association in the Gulf of Oman (high temperatures and normal oceanic salinity water), but its abundance decreased dramatically with the distance to the connecting Strait of Hormuz (Figure 1; Supplementary Figure S2). At the border zone of high-/normal-salinity water bodies (Musandam), the percentage of C3-associated *Porites* spp. dropped to 43%. With increasing distance to Musandam/Strait of Hormuz, the percentage reduced further to 18% (Fujairah) and to 4% in Muscat. At this latter location, 75% of the colonies were associated with C15-type zooxanthellae, the *P. lobata/P. lutea* symbiont usually found elsewhere (Figure 1; Supplementary Figure S2 and Supplementary Table S3). *Symbiodinium* clade D was detected in some *Porites* individuals collected in the transition zone between the PAG and the Gulf of Oman.

**Figure 2:**
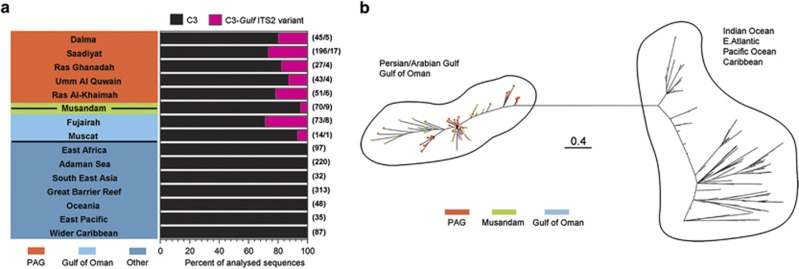
Geographic distribution of *Porites/Symbiodinium* associations. (**a**) Proportion of *Symbiodinium* rDNA ITS2-type C3 and C3*-Gulf ITS2 variant* marker sequences detected in *Porites* spp. corals from within eight locations in the PAG and the Gulf of Oman (Dalma to Muscat, Figure 1a; Supplementary Table S4) and in corals from reference regions. All available reference C3 sequences were retrieved from GeoSymbio (https://sites.google.com/site/geosymbio/database) and GenBank (https://www.ncbi.nlm.nih.gov/genbank/) and included independent of the taxonomic background of the host corals. The number of analysed sequences/number of analysed colonies are shown in brackets next to the bars. (**b**) Phylogenetic tree produced by Bayesian inference analysis of *psb*A^ncr^ sequences from C3-type zooxanthellae samples from the PAG, Musandam and the Gulf of Oman (colour coded as indicated in the legend) and from zooxanthellae of the C3-type and closely related variants from elsewhere (regions detailed in the figure). *psb*A^ncr^ sequences of *S. thermophilum* from the PAG (Hume *et al.*, 2015) were included for reference (open circles). Branches are drawn to scale as indicated by the bar. The separation of the *S. thermophilum* group in the PAG and the Gulf of Oman from the group containing the available sequences from elsewhere in the world is supported with a posterior probability value of 1.0. All accession numbers of the *psb*A^ncr^ sequences used in the analysis are given in Supplementary Tables S5 and S6.

About 23% of the >510 ITS2 sequences of *Porites*-associated *Symbiodinium* subclade C3 contained the C3-*Gulf ITS2 variant* (*sensu* (Hume *et al.*, 2015)) characteristic for *S. thermophilum* (Figure 2a; Supplementary Table S4). This sequence was found in every sampling location in at least ~40% of the analysed *Porites* colonies with C3-type zooxanthellae and also in C3-associated *Porites* colonies in the Gulf of Oman.

Phylogenetic analysis of *psb*A^ncr^ sequences by Bayesian inference was performed for C3-type zooxanthellae samples from the PAG and the Gulf of Oman as well as for those representing the non-Gulf C3-type and closely related variants from elsewhere. Inferred phylogenies resolved the PAG and Gulf of Oman sequences as a well-supported group (PP=1.0) genetically distinct from non-Gulf C3-type sequences, giving average between-group genetic distances of 0.299 (PAG vs non-Gulf) compared with average within-group genetic distances of 0.049 and 0.092 for Gulf ITS2-type C3 and non-Gulf C3 sequences, respectively. The *S. thermophilum* group contains distinct clusters, which may indicate that it comprises of several distinct genetic lineages.

Importantly, this analysis resolved all available *psb*A^ncr^ sequences from C3-type zooxanthellae from the Gulf of Oman (Fujairah and Muscat) and Strait of Hormuz (the tip of the Musandam peninsula) in the same grouping as *S. thermophilum* reference sequences and samples from inside the PAG acquired for the present study (Figure 2b; Supplementary Tables S5 and S6).

With increasing distance to the main distribution area in the southern PAG (100% prevalence), the abundance of *Porites* colonies hosting symbionts of the *S. thermophilum* group decreases steadily along the coast of the Gulf of Oman and from there on becomes essentially undetectable in other parts of the world.

### Thermal tolerance of corals associated with *S. thermophilum* at reduced salinity

Corals have limited tolerance to changes in salinity (Coles, 1992). Since the reduced abundance of *Porites* spp. associated with symbionts of the *S. thermophilum* group in the Gulf of Oman is correlated with a remarkable drop in salinity levels, the question arises whether the prevalence of this association in the southern PAG is related to the exceptionally high-salinity levels of these waters. Therefore, we conducted experiments under controlled laboratory conditions to assess the heat tolerance of *S. thermophilum*-hosting PAG corals at lower-salinity levels found in reefs elsewhere. For this purpose, we cultured replicate colonies of two strains of *P. lobata* originating from Saadiyat reef in the southern PAG (Hume *et al.*, 2013) in our experimental mesocosm (D'Angelo and Wiedenmann, 2012) at salinity levels commonly observed in PAG waters (high salinity, 42) and the adjacent Gulf of Oman (reduced salinity, 36.5) at a constant temperature of 27.5 °C. Both strains host *S. thermophilum* (Supplementary Table S4). Replicate colonies of three species of Indo-Pacific corals (*M. foliosa/Symbiodinium* clade C, *Montipora sp./Symbiodinium* subclade C21/C3u and *P. lichen/Symbiodinium* subclade C21.1/C96) that originated from reefs with normal oceanic salinity levels in the range of 31–34 (D'Angelo and Wiedenmann, 2012) were included as environmental controls in the study. Keeping corals adapted to different native salinity levels together in the respective experimental compartments allowed us to confirm that the regular water exchange between the high- and low-salinity systems sustained comparably good water quality in both set-ups.

**Figure 3:**
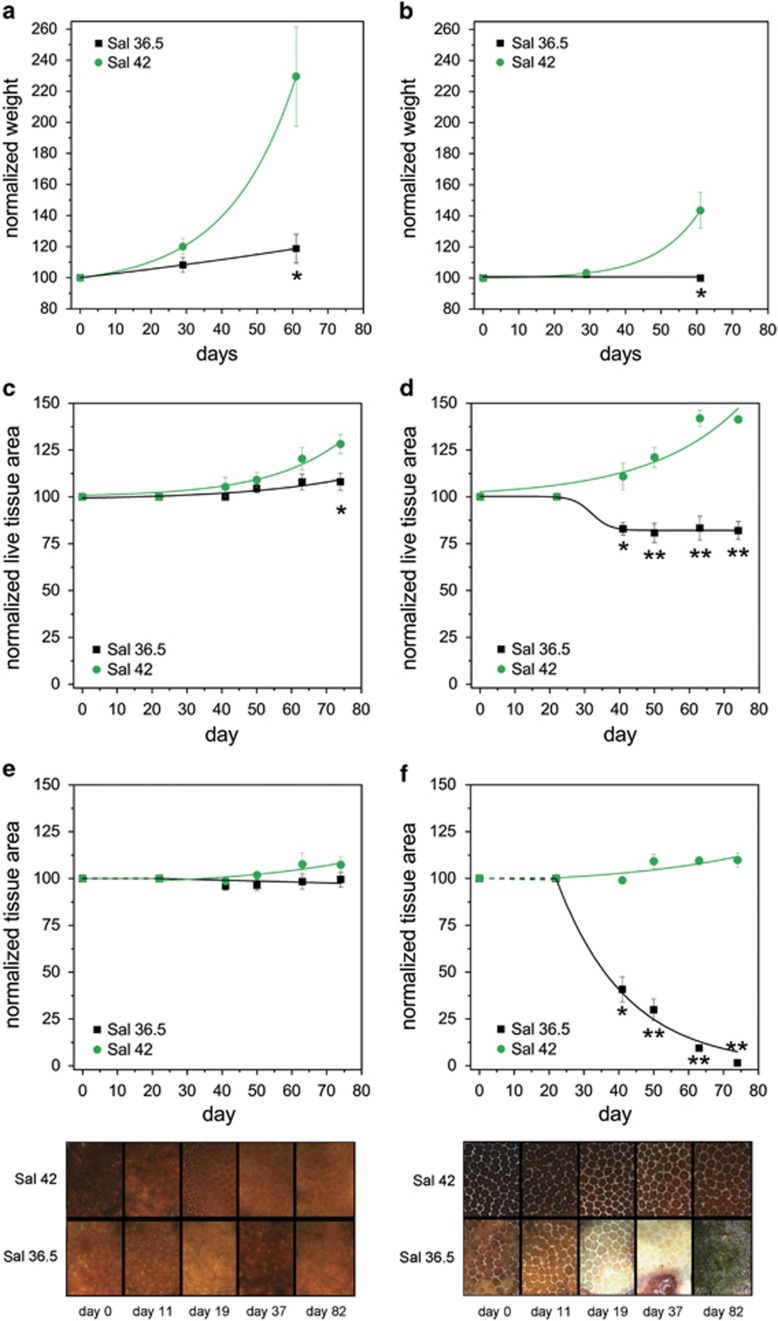
Effect of reduced salinity on the growth of *P. lobata/S. thermophilum* associations from the PAG. Growth of replicate colonies of two strains (designated 1 and 2, left and right panels, respectively) of PAG *P. lobata/S. thermophilum* is shown at high (42) or reduced (36.5) salinity levels. Changes in weight (**a** and **b**) and colony surface area (**c** and **d**) covered by live tissue were determined in independent experiments. The normalised data represent average values of the corresponding replicates in each experiment, bars denote s.e.m. Lines display theoretical model fittings performed as described in Supplementary Table S1. (**e** and **f**) Replicate colonies of PAG *P. lobata/S. thermophilum* (strains 1 and 2) were acclimated to 36.5 or 42 salinity levels at 27.5 °C. At time point 0, temperatures were slowly ramped up (0.5 °C per day) and the corals were cultured at 32 °C for >2 months. Graphs display the changes in colony surface covered by live tissue. The data points represent the average values. Error bars denote s.e.m. Significant values of *P*<0.05 or *P*<0.01 (two sample *t*-test) are indicated with one or two asterisks, respectively, below the corresponding experimental values. After an initial lag phase of ~3 weeks (dashed lines), the data could be fitted (full lines) as described in Supplementary Table S1. Representative micrographs of replicate colonies (lower panels) illustrate the time course of differential responses of strains 1 and 2.

We did not observe obvious changes in the appearance of PAG corals at salinity levels found in the Gulf of Oman during the first 3 weeks of the experiments in the absence of temperature stress (Figures 3a and d). However, prolonged monitoring of changes in colony weight (Figures 3a and b) and in live tissue area (Figures 3c and d) in two independent 5-month experiments showed that, eventually, growth of *P. lobata/S. thermophilum* from the PAG is strongly reduced (strain 1) or stops completely (strain 2) in the low-salinity treatment. In an independent experiment, we exposed PAG *P. lobata/S. thermophilum* acclimated to high and reduced salinity to elevated temperatures of 32 °C, which represents a common summer temperature in the Gulf of Oman as reported by (Coles, 2003). The experimental specimens cultured at lower salinity ceased to grow (strain 1; Figure 3e) or lost 90% of their live tissue after ~63 days and died eventually (strain 2; Figure 3f). In contrast, the corals cultured at 32 °C and high salinity continued to grow as indicated by the increase in live tissue area (Figures 3e and f), although at a slower rate compared to the controls kept at 27.5 °C (Figures 3c and d).

**Figure 4:**
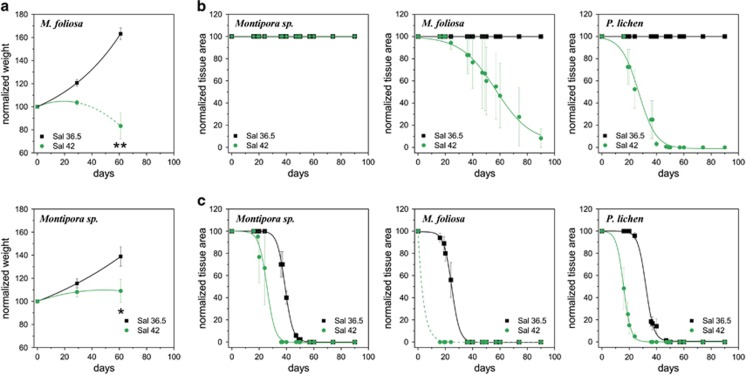
Effect of salinity on the growth of Indo-Pacific corals. (**a**) Replicate colonies of *M. foliosa* and *Montipora sp.* were cultured at high (42) and reduced (36.5) salinity levels for >2 months. The wet weight of each fragment was determined at three time points during the incubation period. The data were normalised to the corresponding initial measurement values. Data points indicate changes in the average weight of five replicate colonies per species and condition, error bars denote s.e.m. Lines correspond to theoretical model fittings as detailed in Supplementary Table S1. The death of *M. foliosa* replicates before the last weight determination is indicated by a dashed line. Asterisks indicate significant difference between the treatments below the corresponding experimental values (two sample *t*-test, *P<0.05 and **P<0.01). (**b** and **c**) Effects of salinity and temperature stress on the survival of Indo-Pacific corals. Replicate colonies of *M. foliosa*, *Montipora sp.* and *P. lichen* corals were acclimated to two salinity levels (42 and 36.5) at a constant temperature of 27.5 °C. The temperature remained unaltered for the control corals over the duration of the >3-month experiment (**b**). At time point 0, temperatures were slowly ramped up (0.5 °C per day) for the treatment group and corals were incubated at 32 °C until the end of the experiment (**c**). The graphs display the changes in colony surface covered by live tissue. The data points represent the average of four and five colonies per genotype for the control and temperature treatments, respectively. Error bars denote s.e.m. Lines correspond to the theoretical models as detailed in Supplementary Table S1. *M. foliosa* samples exposed to Sal 42 under high-temperature stress died before the 16-day measurements, which is indicated by the dashed line section of the corresponding fitting.

The Indo-Pacific corals exhibited a contrasting behaviour to PAG specimens in these parallel treatments. They showed exponential growth under low-salinity conditions (Figures 4a and b), however, they ceased to grow or started to die after ~4 weeks at ambient temperature (27.5 °C) in the high-salinity treatment (Figures 4a and b). At elevated temperatures (32 °C), complete mortality of specimens kept at high salinity occurred ~2–3 weeks earlier compared to the treatment during which replicate colonies of the three species were exposed to high temperature at a salinity of 36.5 (Figure 4c). Under the latter conditions, the corals lost 90% of their live tissue in about 30–43 days. *P. lobata–Symbiodinium* C15 from Fiji has shown a comparable susceptibility to heat stress in a similar experiment (Hume *et al.*, 2013).

## Discussion

### Distribution of the *S. thermophilum* group

Our present analysis reveals a striking predominance of *Porites spp.* symbioses with members of the *S. thermophilum* group in the southern PAG. These associations were found also in the Gulf of Oman, albeit at lower abundance. The steep decline in the frequency of C3-associated *Porites* spp. along the temperature and salinity gradient between the two waterbodies (Figure 1; Supplementary Figure S2) indicates that the high-salinity waters of the southern PAG represent the main distribution area of this unusual *Porites–Symbiodinium* symbiosis. In contrast, *P. lobata* and *P. lutea* populations from elsewhere in the world harbour almost exclusively *Symbiodinium* belonging to C15 cluster (rDNA ITS2 type). These findings suggest that the symbiosis with zooxanthellae of the *S. thermophilum* group is essential for coral survival in the extreme temperature and salinity environment of the southern PAG.

An uncommon association of *Porites* with *Symbiodinium* clade D was detected in some individuals collected in the transition zone between the PAG and the Gulf of Oman. Although some members of this symbiont clade are considered to increase the thermal tolerance of the holobiont among other coral species (Baker *et al.*, 2004), our results show that it is not the basis for the thermal tolerance of *Porites* spp. under the prevailing environmental conditions of the southern PAG.

The gradual decline in the abundance of *Porites* spp./*S. thermophilum* group associations outside the PAG and the increasing dominance of the ‘cosmopolitan' *Porites*–C15 associations indicate that the *Porites/S. thermophilum* partnership is—at least at the level of the holobiont—less successful outside of the extreme environment of the southern PAG. This is despite the fact that the temperature regime in the Gulf of Oman with large fluctuations and regular summer peaks of 32 °C is more challenging compared to most reef regions elsewhere (Riegl *et al.*, 2011). Therefore, survival in the extreme environment of the PAG depends not only on temperature stress tolerance but also on the adaptation of multiple traits to local conditions that can constrain the physiological performance of the symbiotic association in other habitats. The change between the extraordinarily high salinity of the PAG and the normal oceanic salinity of the Gulf of Oman is well correlated with the decreasing prevalence of the association of *Porites* spp. with members of the *S. thermophilum* group, suggesting that salinity levels might have an important influence on the local adaptation of coral–alga symbioses. Indeed, we observed impaired growth and reduced heat tolerance of *P. lobata/S. thermophilum* associations at experimentally reduced salinity levels. Our findings suggest that the *P. lobata/S. thermophilum* symbiosis from the PAG is strongly adapted not only to the elevated temperatures but also to the exceptionally high salinity that characterises their local environment.

However, it is critical to note that although impaired by the reduced salinity, *P. lobata/S. thermophilum* endured heat stress for a longer period of time compared to the three species of Indo-Pacific corals tested in the present study and to *P. lobata/Symbiodinium* C15 from Fiji (Hume *et al.*, 2013).

At present, it is not possible to discern whether local adaptation to temperature and salinity is dominated by physiological responses of the coral host or those of the algal symbionts. Although the host is likely to play an important role (Baird *et al.*, 2009a), the prominent presence of *S. thermophilum* in the southern PAG (Hume *et al.*, 2013; Hume *et al.*, 2015) suggests that the symbionts make a decisive contribution. In fact, the only hint to a more widespread distribution of the *S. thermophilum* group exists in the form of a single database entry that reports a C3-*Gulf ITS2 variant* sequence from *Stylophora pistillata* from the Red Sea, an environment where temperatures and salinity can also reach exceptionally high levels on a regular basis (Hume *et al.*, 2015). The importance of local adaptations of *Symbiodinium* types for the thermal tolerance of the coral holobiont has been also demonstrated for *Acropora millepora* from the Great Barrier Reef in Australia (Howells *et al.*, 2012).

In agreement with the lower abundances of associations of *Porites* spp. with symbionts of the *S. thermophilum* group in the Gulf of Oman, our experiments confirmed that some corals associated with *S. thermophilum* can survive at normal oceanic salinity levels. However, the trade-offs resulting from local adaptation to deviating environmental conditions can strongly impair their physiological performance and offer a likely explanation for the constrained distribution of these symbiotic associations.

### Implications for coral reef future and coral reef management

Despite the geographic isolation expected for PAG organisms (Sheppard *et al.*, 1992), our results demonstrate that the distribution of symbionts of the *S. thermophilum* group, which are integral to coral survival in the world's hottest seas, is not restricted to this water body. Instead, it can also be found in the adjacent Gulf of Oman where regular peak summer temperatures of 32 °C select for temperature stress-tolerant genotypes (Coles and Riegl, 2013; Riegl *et al.*, 2012). The distribution across the Strait of Hormuz could potentially be promoted by the minor surface outflow of the PAG (Johns *et al.*, 1999) that extends along the coast of Oman (Figure 1a) and runs in the opposite direction as the dominant inflow current (Sheppard *et al.*, 1992). The Gulf of Oman might thus represent a ‘melting pot' where traits such as temperature and salinity tolerance could be recombined, potentially producing genotypes that might be preadapted to the warmer oceans of the future. Global connectivity models (Wood *et al.*, 2014) suggest that coral larvae with vertically transmitted symbionts (Baird *et al.*, 2009b) might carry this genetic material from the Gulf of Oman into the Indian Ocean. Therefore, a high priority should be assigned to the protection of the marine environment in the PAG (Sale *et al.*, 2011) and the Gulf of Oman.

Corals from the PAG have been discussed as subjects for assisted migration projects to protect them from the threat of rising temperature in their original habitat and to help to replenish reefs devastated elsewhere by global warming with heat-tolerant genotypes (Coles and Riegl, 2013). In addition to the general concerns about the feasibility and risks of long-range translocation and introduction of organisms into non-native environments (Coles and Riegl, 2013), our results suggest that due to the strong local adaptation, the superior stress tolerance of coral/*S. thermophilum* associations from the PAG might be reduced or even lost when they are exposed to the lower-salinity levels commonly found in the majority of reefs elsewhere, accordingly reducing the value of this conservation approach. Moreover, the weaker performance at lower salinity might contribute to an outbreeding depression (Aitken and Whitlock, 2013) in recipient populations. Further research is desirable to establish how other PAG coral/symbiont species would respond to changes in salinity. Meanwhile, coral reef management should not rely on long-range-assisted migration as a generally applicable solution for reefs devastated by global warming, but rather concentrate on alternative actions to strengthen the resilience of reef ecosystems.
