# Supplementary material for: Local adaptation constrains the distribution potential of heat-tolerant Symbiodinium from the Persian/Arabian Gulf
Source: ISME J. 2015 May 19;9(12):2551–60. doi: 10.1038/ismej.2015.80 (PMC4817622; doi:10.1038/ismej.2015.80)
Supplement: Supplementary Information [file ismej201580x1.doc]

**Local adaptation constrains the distribution potential of heat tolerant *Symbiodinium* from the Persian/Arabian Gulf**

Cecilia D’Angelo^1*^, Benjamin C.C. Hume^1*^, John Burt^2^, Edward G. Smith^2^,

Eric P. Achterberg^1,3^ and Jörg Wiedenmann^1,4^

*equal contribution

^1^ Coral Reef Laboratory. Ocean and Earth Science, University of Southampton, Waterfront Campus, National Oceanography Centre, Southampton, SO14 3ZH, UK

^2^ Marine Biology Laboratory, Centre for Genomics and Systems Biology, New York University – Abu Dhabi, PO Box 129 188, Abu Dhabi, United Arab Emirates

^3^ GEOMAR, Helmholtz Centre for Ocean Research, 24148 Kiel, Germany

^4^ Institute for Life Sciences, University of Southampton, Highfield Campus, Southampton, SO17 1BJ, UK

**Supplementary Information**

**Supplementary methods**

DGGE analysis

The methodology utilised is based on a modification of previously described protocols . PCR amplification for DGGE analysis was achieved by using 0.5µM of each primer SYM_VAR_5.8SII 5’-ATCTTGGCTCGAGCACCTATGAAGG-3’ (priming in the 5.8S region of the ITS2 rRNA gene) and SYM_VAR_Clamp (identical to SYM_VAR_REV with the addition of a 40bp GC clamp) 5’ [CGCCCGCCGCGCCCCGCGCCCGT­CCCGCCGCCCCCGCCC] CGGGTTCTCTTG TTTGACTTCATGC3’ in a reaction mixture with 200µM of each dNTP, 2.5mM MgCl2, 1X GoTaq Flexi buffer, 2.5U of GoTaq DNA Polymerase and ~50 ng of template DNA. PCRs were performed using a MyCycler thermocycler (BioRad) with an initial denaturation step (95ºC / 30 s) followed by 35 cycles of 95ºC/30 s, 56ºC/30 s, 72ºC/30 s with a final extension step of 72ºC/5 mins.

DGGE analyses were conducted using a BioRad DCode System for DGGE with a model 475 gradient former. Samples were run on 8% polyacrylamide gels with a gradient of 35-65%. The 100% denaturant solution contained 40% (v/v) of formamide (deionized) and 42% (w/v) of urea. Immediately before pouring, the denaturant solutions were degassed using a sonic water bath for 3 minutes under vacuum at 65ºC. When using a 16 well gel comb, samples were run at 150V for 3 h and 10µl of PCR product was loaded with 10µl of 2x gel loading dye; with the 32 well comb, samples were run at 130V for 2.5 h and 5µl of PCR sample was loaded with 5µl of 2x gel loading dye. Gels were stained for 15 min in 200 ml of TAE running buffer with a final ethidium bromide concentration of 50 µg/ml and destained afterwards in water for a further 15 min. Markers for ITS2 phylotypes of distinct clades were created by amplifying relevant fragments from cloned plasmid DNA templates that contained previously sequenced and characterised 18S-ITS1-5.8S-ITS2-28S rRNA genes including those of our previous study . When producing these DNA markers, the annealing temperature of the PCR was raised to 66ºC and the number of cycles was reduced to 30.

The dominant *Symbiodinium* ITS2 subcladal type was identified for each sample. Prominent bands were excised from gels using a sterile scalpel, placed in 500µl of water and homogenised with a micropestle before being incubated at 4^o^C overnight. After incubation, samples were centrifuged at maximum speed for one minute and 4µl of this supernatant was used as template in a further PCR using SYM_VAR_5.8SII and SYM_VAR_REV primers using Advantage 2 Polymerase mix (Clontech) as described above. The PCR protocol included 95^o^C for 1 min followed by 30 cycles of 95^o^C for 30 s, 56^o^C for 30 s and 68^o^C for 30 s and by a final extension of 2 min at 68^o^C. The PCR products were cloned and sequenced as detailed above. When samples yielded characteristic multi band patterns, representative bands were excised, the contained DNA re-amplified using the SYM_VAR_5.8SII /SYM_VAR_Clamp primers and re-run on a DGGE gel. If these amplicons ran with an identical “fingerprint” to the original, the pattern was attributed to a clade / sequence-specific formation of DNA structures and no further analysis was undertaken. Otherwise, the sequences of the relevant fragments were determined as described above.

The DGGE procedure developed in this study was successfully applied to distinguish among important *Symbiodinium* ITS2 phylotypes (clades A, D1, C15 and C3) with a resolution comparable to previous studies (e.g.). Importantly, meaningful results were obtained already after the relatively short gel running time of 2.5-3h (as compare to >9 h, i.e. ). Contamination with host DNA can be a confounding problem in DGGE studies of zooxanthellae phylotypes . The use of re-designed primers to specifically differentiate between *Porites* host and *Symbiodinium* DNA has proved successful with no host-derived sequence being identified during our study.

**Supplementary References**

Hume B, D’Angelo C, Burt J, Baker A, Riegl B, Wiedenmann J (2013). Corals from the Persian/Arabian Gulf as models for thermotolerant reef-builders: Prevalence of clade C3 *Symbiodinium*, host fluorescence and *ex situ* temperature tolerance. *Mar Pollut Bull* **72:** 313-322.

LaJeunesse T (2002). Diversity and community structure of symbiotic dinoflagellates from Caribbean coral reefs. *Mar Biol* **141:** 387-400.

Thornhill DJ, Lajeunesse TC, Santos SR (2007). Measuring rDNA diversity in eukaryotic microbial systems: how intragenomic variation, pseudogenes, and PCR artifacts confound biodiversity estimates. *Mol Ecol* **16:** 5326-5340.

Thornhill DJ, Kemp DW, Sampayo EM, Schmidt GW (2010). Comparative analyses of amplicon migration behavior in differing denaturing gradient gel electrophoresis (DGGE) systems. *Coral Reefs* **29:** 83-91.

**Supplementary Figures**

**
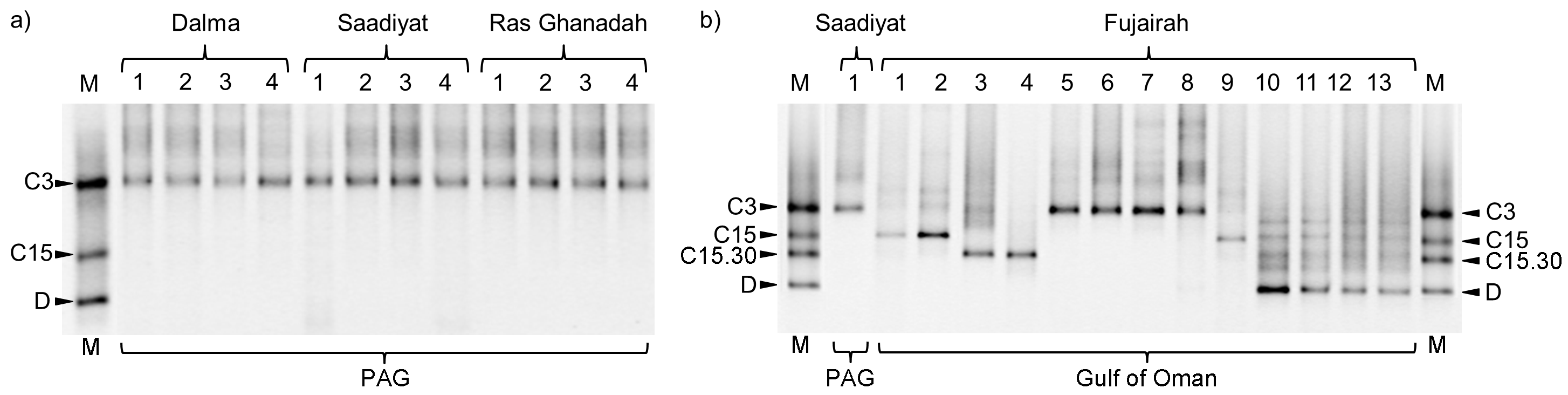
**

**Supplementary Figure S1**: **Representative analytical denaturing gradient gel electrophoresis (DGGE) gels of the internal transcribed spacer 2 (ITS2) region of the *Symbiodinium spp****.* The predominant ITS2 ‘type’ *Symbiodinium* harboured by *P. lutea* and *P. lobata* was determined in different locations from the PAG (a) and the Gulf of Oman (b). One sample collected in Saadiyat is included in b) (as indicated) for reference. Reference marker sequences (C3, C15-cluster, D) are run on each of the gels (black arrows) and annotated according to the ITS2 type sequence they represent.


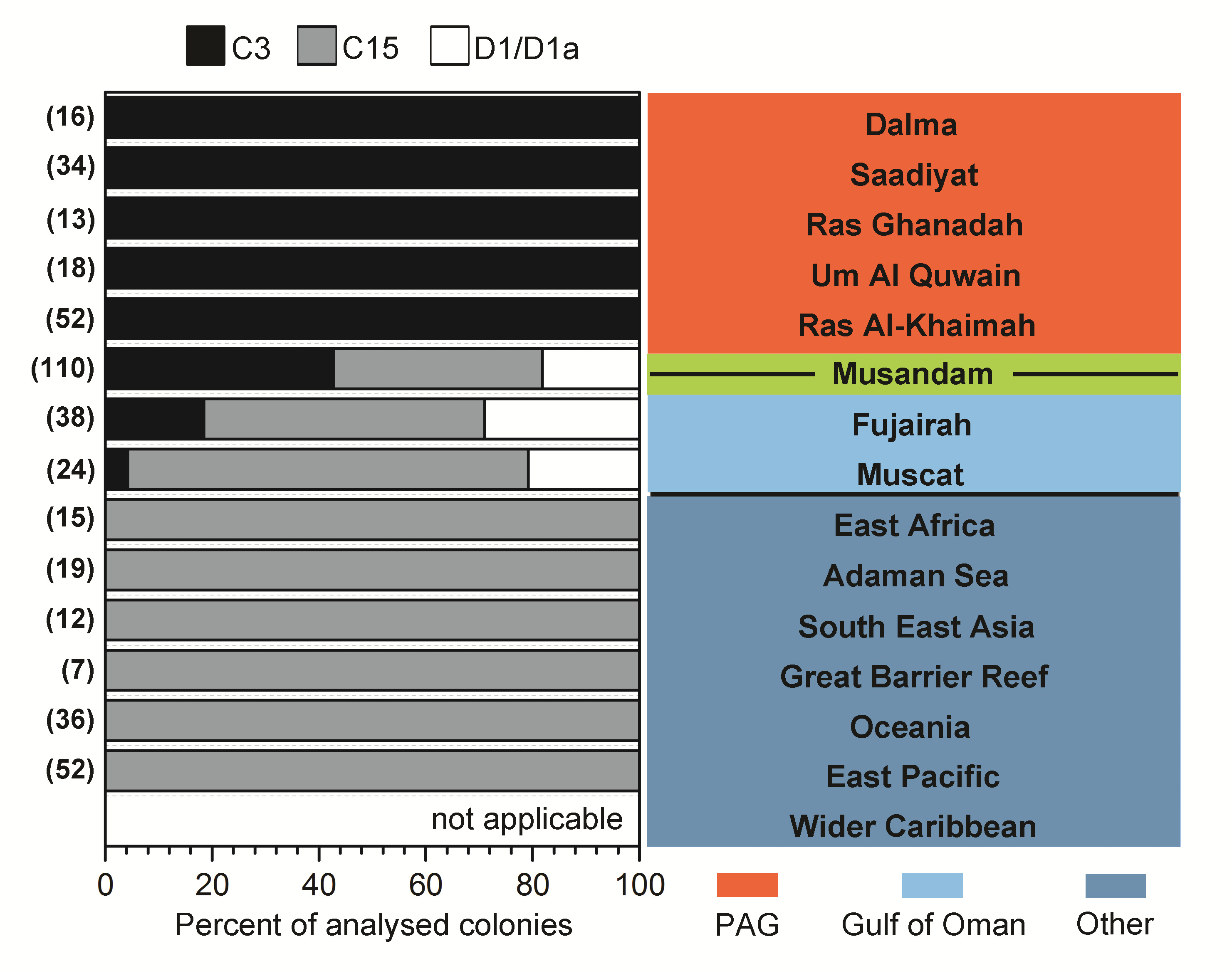


**Supplementary Figure S2: Geographic distribution of *Porites* - *Symbiodinium* associations.** The dominant *Symbiodinium* clades associated with individual colonies of *P. lobata, P. lutea and P. harrisoni* within eight locations in the PAG and the Gulf of Oman (Dalma to Muscat, classified by DGGE analysis of rDNA ITS2) and *P. lobata and P. lutea* from six reference regions (East Africa to Caribbean, literature-based meta-analysis detailed in Fig. 1b and Table S3). The number of independent coral colonies analysed for each location is indicated in brackets.

**Supplementary Tables**

| **Curve Fit** | **Equation** | | **Species** | | **Parameter** | **Treatment** | **Sal** | **Fig.** |
| --- | --- | --- | --- | --- | --- | --- | --- | --- |
| Exponential | 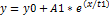 | | *P. lobata* strain 1 | | weight | control | 36 | 3 |
|  |  |  | *P. lobata* strain 1 | | weight | control | 42 | 3 |
|  |  |  | *P. lobata* strain 2 | | weight | control | 42 | 3 |
|  |  |  | *P. lobata* strain 1 | | tissue area | control | 36 | 3 |
|  |  |  | *P. lobata* strain 1 | | tissue area | control | 42 | 3 |
|  |  |  | *P. lobata* strain 2 | | tissue area | control | 42 | 3 |
|  |  |  | *P. lobata* strain 1 | | tissue area | stress | 42 | 3 |
|  |  |  | *P. lobata* strain 2 | | tissue area | stress | 42 | 3 |
|  |  |  | *M. foliosa* | | weight | control | 36 | 4 |
|  |  |  | *Montipora sp* | | weight | control | 36 | 4 |
| Linear | 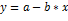 | | *P. lobata* strain 2 | | weight | control | 36 | 3 |
|  |  |  | *P. lobata* strain 1 | | tissue area | stress | 36 | 3 |
|  |  |  | *M. foliosa* | | tissue area | control | 36 | 4 |
|  |  |  | *Montipora sp* | | tissue area | control | 36 | 4 |
|  |  |  | *Montipora sp* | | tissue area | control | 42 | 4 |
|  |  |  | *P. lichen* | | tissue area | control | 36 | 4 |
| Sigmoid | 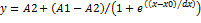 | | *P. lobata* strain 2 | | tissue area | control | 36 | 3 |
|  |  |  | *M. foliosa* | | tissue area | control | 42 | 4 |
|  |  |  | *M. foliosa* | | tissue area | stress | 36 | 4 |
|  |  |  | *Montipora sp* | | tissue area | stress | 36 | 4 |
|  |  |  | *Montipora sp* | | tissue area | stress | 42 | 4 |
|  |  |  | *P. lichen* | | tissue area | control | 42 | 4 |
|  |  |  | *P. lichen* | | tissue area | stress | 36 | 4 |
|  |  |  | *P. lichen* | | tissue area | stress | 42 | 4 |
| Exponential | 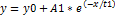 | *P. lobata* strain 2 | | tissue area | | stress | 36 | 3 |
|  |  | | *M. foliosa* | | tissue area | stress | 42 | 4 |
| polynomial | 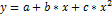 | | *M. foliosa* | | weight | control | 42 | 3 |
|  |  |  | *Montipora sp* | | weight | control | 42 | 3 |

**Supplementary Table S1: Theoretical model fit of experimental data.**

| **Site** | **Species** | **# Colonies with dominant ITS2 type:** | | | |
| --- | --- | --- | --- | --- | --- |
|  |  | **C3** | **C15** | **C15.30** | **D** |
| Dalma | *P. lutea* | 4 | -- | -- | -- |
|  | *P. harrisoni* | 12 | -- | -- | -- |
| Saadiyat | *P. lobata* | 12 | -- | -- | -- |
|  | *P. lutea* | 11 | -- | -- | -- |
|  | *P. harrisoni* | 11 | -- | -- | -- |
| Ras Ghanadah | *P. lobata* | 3 | -- | -- | -- |
|  | *P. lutea* | 5 | -- | -- | -- |
|  | *P. harrisoni* | 5 | -- | -- | -- |
| Umm Al Quwain | *P. lutea* | 18 | -- | -- | -- |
| Ras Al-Khaimah | *Porites sp.* | 52 | -- | -- | -- |
| Musandam | *P. lobata* | 28 | 21 | 6 | 7 |
|  | *P. lutea* | 13 | 7 | 3 | 6 |
|  | *P. harrisoni* | 6 | 4 | 2 | 7 |
| Fujairah | *P. lobata* | 6 | 12 | 4 | 7 |
|  | *P. lutea* | 1 | 2 | 2 | 2 |
|  | *P. harrisoni* | -- | -- | -- | 2 |
| Muscat | *Porites sp.* | 1 | 9 | 9 | 5 |
|  |  |  |  |  |  |

**Supplementary Table S2: Dominant *Symbiodinium* clades in *Porites* spp. colonies as identified by DGGE of the ITS2 region.**

**Supplementary Table S3: References for *Porites lobata / lutea – Symbiodinium* associations.**

| **Region number and location** | **Species** | **Colonies** | **Phylotype** | **Reference** |
| --- | --- | --- | --- | --- |
| **1 Johnston Atoll (EP)** | *P. lobata* | 5 | C15-cluster | 1 |
| **2 Oahu, Hawaii (EP)** | *P. lobata* | 4 | C15-cluster | 2 |
|  | *P. lobata* | 76 | G3(4) C15-Cluster (72) | 3 |
|  | *P. lutea* | 3 | C15-cluster | 2 |
| **3 Palmyra Atoll (EP)** | *P. lobata* | 2 | C15-cluster | 4 |
| **4 Moorea, French Polynesia (EP)** | *P. lobata* | 5 | C15-cluster | 5 |
|  | *P. lutea* | 5 | C15-cluster | 5 |
|  | *P. lutea* | 13 | C15-cluster | 6 |
| **5 Darwin Island, Galapagos**  **Archipelago (EP)** | *P. lobata* | 15 | C15-cluster | 7 |
| **6 Zanzibar, Tanzania (EA)** | *P. lobata* | 6 | C15-cluster | 8 |
|  | *P. lutea* | 9 | C15-cluster | 8 |
| **7 Cape Panwa, Thailand (AS)** | *P. lobata* | 5 | C15-cluster | 8 |
|  | *P. lutea* | 14 | C15-cluster | 8 |
| **8 Halong Bay, Vietnam (SEA)** | *P. lutea* | 4 | C15-cluster | 9 |
| **9 Hainan, China Sea (SEA)** | *P. lutea* | 4 | C15-cluster | 10 |
| **10 Kenting National Park, Taiwan (SEA)** | *P. lutea* | 1 | C15-cluster | 11 |
| **11 Okinawa, Japan (SEA)** | *P. lutea* | 3 | C15-cluster | 12 |
| **12 Heron Island, Australia (GBR)** | *P. lutea* | 7 | C15-cluster | 13 |
| **13 Bega, Fiji (OC)** | *P. lobata* | 3 | C15-cluster | 14 |
| **14 Ofu, American Samoa (OC)** | *P. lobata* | 6 | C15-cluster | 15 |
|  | *P. lobata* | 27 | C15-cluster | 16 |

Regional numbers refer to within chart integers in Figure 1b. Abbreviations in parentheses correspond to the six reference regions in Suppl Fig. S2: EA – East Africa; AS – Andaman Sea; SEA – Southeast Asia; GBR – Great Barrier Reef, Australia; OC – Oceania; EP – East Pacific.

1. Stat, M., Pochon, X., Cowie, R. & Gates, R. Specificity in communities of *Symbiodinium* in corals from Johnston Atoll. *Mar. Ecol. Prog. Ser.* **386,** 83–96 (2009).
2. Apprill, A. M. & Gates, R. D. Recognizing diversity in coral symbiotic dinoflagellate communities. *Mol. Ecol.* **16,** 1127–34 (2007).
3. Stat, M. *et al.* The distribution of the thermally tolerant symbiont lineage (*Symbiodinium* clade D) in corals from Hawaii: correlations with host and the history of ocean thermal stress. *Ecol. Evol.* **3,** 1317–29 (2013).
4. Wicks, L. C., Gardner, J. P. A. & Davy, S. K. Host tolerance, not symbiont tolerance, determines the distribution of coral species in relation to their environment at a Central Pacific atoll. *Coral Reefs* **31,** 389–398 (2011).
5. Putnam, H. M., Stat, M., Pochon, X. & Gates, R. D. Endosymbiotic flexibility associates with environmental sensitivity in scleractinian corals. *Proc. Biol. Sci.* **279,** 4352–61 (2012).
6. Edmunds, P. J., Putnam, H. M. & Gates, R. D. Photophysiological consequences of vertical stratification of Symbiodinium in tissue of the coral Porites lutea. *Biol. Bull.* **223,** 226–235 (2012).
7. Glynn, P. W., Riegl, B., Correa, A. M. S. & Baums, I. B. Rapid recovery of a coral reef at Darwin Island, Galápagos Islands. *Galápagos Res* **66,** 6–13 (2009).
8. LaJeunesse, T. C. *et al.* Long-standing environmental conditions, geographic isolation and host-symbiont specificity influence the relative ecological dominance and genetic diversification of coral endosymbionts in the genus *Symbiodinium*. *J. Biogeogr.* **37,** 785–800 (2010).
9. Faxneld, S. Coral reefs in the Anthropocene : The effects of stress on coral metabolism and symbiont composition. *PhD thesis. Stock. Univ.* (2011). at <http://www.diva-portal.org/smash/record.jsf?pid=diva2:445323>
10. Zhou, G.-W. & Huang, H. Low genetic diversity of symbiotic dinoflagellates (*Symbiodinium*) in scleractinian corals from tropical reefs in southern Hainan Island, China. *J. Syst. Evol.* **49,** 598–605 (2011).
11. Wang, J. T., Meng, P. J., Chen, Y. Y. & Chen, C. A. Determination of the thermal tolerance of *Symbiodinium* using the activation energy for inhibiting photosystem II activity. *Zool. Stud.* **51,** 137–142 (2012).
12. LaJeunesse, T. C. *et al.* Closely related *Symbiodinium spp.* differ in relative dominance in coral reef host communities across environmental, latitudinal and biogeographic gradients. *Mar. Ecol. Prog. Ser.* **284,** 147–161 (2004).
13. Fisher, P. L., Malme, M. K. & Dove, S. The effect of temperature stress on coral–*Symbiodinium* associations containing distinct symbiont types. *Coral Reefs* **31,** 473–485 (2012).
14. Hume, B. *et al.* Corals from the Persian/Arabian Gulf as models for thermotolerant reef-builders: prevalence of clade C3 *Symbiodinium*, host fluorescence and *ex situ* temperature tolerance. *Mar. Pollut. Bull.* **72,** 313–22 (2013).
15. Smith, L. W., Wirshing, H. H., Baker, a. C. & Birkeland, C. Environmental versus genetic influences on growth rates of the corals *Pocillopora eydouxi* and *Porites lobata* (Anthozoa: Scleractinia). *Pacific Sci.* **62,** 57–69 (2008).
16. Barshis, D. J. et al. Protein expression and genetic structure of the coral *Porites lobata* in an environmentally extreme Samoan back reef: does host genotype limit phenotypic plasticity? *Mol. Ecol.* **19**, 1705-1720, doi:10.1111/j.1365-294X.2010.04574.x (2010).

**Supplementary Table S4: Detection frequency of *Symbiodinium* C3 and the C3-*Gulf* *ITS2 variant* sequences in colonies previously determined by DGGE analyses to contain ITS2-type C3 as dominant symbiont strain.**

| **Region** | **Species** | | **ID** | **Collection** | | | | **Colony #** | | | **Sequences** | | | **C3 *Gulf*:C3** | |
| --- | --- | --- | --- | --- | --- | --- | --- | --- | --- | --- | --- | --- | --- | --- | --- |
| Dalma† | *P. harrisoni* | | BH772 | 9/12 | | | 1 | | | 9 | | | 0:9 | |  |
| Dalma† | *P. harrisoni* | | BH776 | 9/12 | | | 2 | | | 9 | | | 0:9 | |  |
| Dalma† | *P. harrisoni* | | BH1448 | 9/12 | | | 3 | | | 9 | | | 3:6 | |  |
| Dalma† | *P. harrisoni* | | BH1411 | 9/12 | | | 4 | | | 9 | | | 3:6 | |  |
| Dalma† | *P. harrisoni* | | BH787 | 9/12 | | | 5 | | | 9 | | | 3:6 | |  |
| Saadiya†t | *P. lobata* | | BH369 | 9/12 | | | 1 | | | 15 | | | 2:13 | |  |
| Saadiyat †^a)^ | *P. lobata* (Strain 1) | | BH143 | 1/14 | | | 2 | | | 17 | | | 3:14 | |  |
| Saadiyat †^a)^ | *P. lobata* (Strain 2) | | BH164 | 1/14 | | | 3 | | | 13 | | | 3:10 | |  |
| Saadiyat †^a)^ | *P. lobata* | | BH173 | 1/14 | | | 4 | | | 9 | | | 2:7 | |  |
| Saadiyat †^a)^ | *P. lobata* | | BH167 | 1/14 | | | 5 | | | 9 | | | 2:7 | |  |
| Saadiyat† | *P. lobata* | | BH213 | 10/11 | | | 6 | | | 12 | | | 2:10 | |  |
| Saadiyat† | *P. lobata* | | BH218 | 10/11 | | | 7 | | | 7 | | | 1:6 | |  |
| Saadiyat† | *P. lobata* | | BH223 | 10/11 | | | 8 | | | 12 | | | 2:10 | |  |
| Saadiyat† | *P. lobata* | | BH1217 | 9/12 | | | 9 | | | 6 | | | 1:5 | |  |
| Saadiyat† | *P. lobata* | | BH1222 | 9/12 | | | 10 | | | 2 | | | 1:1 | |  |
| Saadiyat† | *P. harrisoni* | | BH313 | 10/11 | | | 1 | | | 3 | | | 1:2 | |  |
| Saadiyat† | *P. harrisoni* | | BH318 | 10/11 | | | 2 | | | 8 | | | 1:7 | |  |
| Saadiyat† | *P. harrisoni* | | BH377 | 9/12 | | | 3 | | | 28 | | | 18:10 | |  |
| Saadiyat† | *P. harrisoni* | | BH386 | 9/12 | | | 4 | | | 28 | | | 16:12 | |  |
| Saadiyat† | *P. harrisoni* | | BH403 | 9/12 | | | 5 | | | 8 | | | 0:8 | |  |
| Saadiyat† | *P. harrisoni* | | BH236 | 10/11 | | | 6 | | | 7 | | | 2:5 | |  |
| Saadiyat† | *P. lutea* | | BH1422 | 9/12 | | | 1 | | | 9 | | | 7:2 | |  |
| Saadiyat† | *P. lutea* | | BH383 | 9/12 | | | 2 | | | 3 | | | 0:3 | |  |
| Ras Ghanadah | *P. lobata* | | BH1138 | 9/12 | | | 1 | | | 7 | | | 2:5 | |  |
| Ras Ghanadah | *P. lobata* | | BH1159 | 9/12 | | | 2 | | | 7 | | | 2:5 | |  |
| Ras Ghanadah | *P .lutea* | | BH1145 | 9/12 | | | 1 | | | 6 | | | 0:6 | |  |
| Ras Ghanadah | *P. lutea* | | BH1152 | 9/12 | | | 2 | | | 7 | | | 1:6 | |  |
| Umm Al Quwain† | *P. lutea* | | BH796 | 3/13 | | | 3 | | | 8 | | | 0:8 | |  |
| Umm Al Quwain† | *P. lutea* | | BH755 | 3/13 | | | 4 | | | 9 | | | 3:6 | |  |
| Umm Al Quwain† | *P. lutea* | | BH762 | 3/13 | | | 5 | | | 18 | | | 3:15 | |  |
| Umm Al Quwain† | *P. lutea* | | BH767 | 3/13 | | | 6 | | | 8 | | | 0:8 | |  |
| Ras Al-Khaimah | *Porites spp.*^b)^ | | BH921 | 3/13 | | | 1 | | | 8 | | | 3:5 | |  |
| Ras Al-Khaimah | *Porites spp.*^b)^ | | BH908 | 3/13 | | | 2 | | | 8 | | | 3:5 | |  |
| Ras Al-Khaimah | *Porites spp.*^b)^ | | BH906 | 3/13 | | | 3 | | | 9 | | | 1:8 | |  |
| Ras Al-Khaimah | *Porites spp.*^b)^ | | BH940 | 3/13 | | | 4 | | | 9 | | | 1:8 | |  |
| Ras Al-Khaimah | *Porites spp.*^b)^ | | BH864 | 3/13 | | | 5 | | | 9 | | | 2:7 | |  |
| Ras Al-Khaimah | *Porites spp.*^b)^ | | BH931 | 3/13 | | | 6 | | | 8 | | | 1:7 | |  |
| Musandam | *P. lobata* | | BH883 | 3/13 | | | 1 | | | 9 | | | 0:9 | |  |
| Musandam | *P. lobata* | | BH1166 | 3/13 | | | 2 | | | 7 | | | 0:7 | |  |
| Musandam | *P. lobata* | | BH1179 | 3/13 | | | 3 | | | 6 | | | 1:5 | |  |
| Musandam | *P. harrisoni* | | BH1173 | 3/13 | | | 1 | | | 6 | | | 0:6 | |  |
| Musandam | *P. harrisoni* | | BH708 | 3/13 | | | 2 | | | 9 | | | 1:8 | |  |
| Musandam | *P. harrisoni* | | BH888 | 3/13 | | | 3 | | | 9 | | | 0:9 | |  |
| Musandam | *P. lutea* | | BH709 | 3/13 | | | 1 | | | 9 | | | 0:9 | |  |
| Musandam | *P. lutea* | | BH718 | 3/13 | | | 2 | | | 9 | | | 0:9 | |  |
| Musandam | *P. lutea* | | BH1185 | 3/13 | | | 3 | | | 6 | | | 1:5 | |  |
| Fujairah | *P. lobata* | | BH737 | 9/12 | | | 1 | | | 8 | | | 7:1 | |  |
| Fujairah | *P. lobata* | | BH753 | 9/12 | | | 2 | | | 8 | | | 0:8 | |  |
| Fujairah | *P. lobata* | | BH738 | 9/12 | | | 3 | | | 8 | | | 5:3 | |  |
| Fujairah | *P. lobata* | | BH806 | 9/12 | | | 4 | | | 10 | | | 0:10 | |  |
| Fujairah | *P. lobata* | | BH957 | 9/12 | | | 5 | | | 10 | | | 6:4 | |  |
| Fujairah | *P. lobata* | | BH963 | 9/12 | | | 6 | | | 10 | | | 2:8 | |  |
| Fujairah | *P. lutea* | | BH740 | 9/12 | | | 1 | | | 9 | | | 0:9 | |  |
| Fujairah | *P. lutea* | | BH973 | 9/12 | | | 2 | | | 10 | | | 0:10 | |  |
| Muscat | *Porites spp.*^b)^ | | BH839 | 3/13 | | | 1 | | | 14 | | | 1:13 | |  |
| **Total** |  |  | | |  | **55** | | | **519** | | | **119:400** | | | |

^a)^ After 3.7 years of aquarium culture, ^b)^ Field-identified as *P. lobata / lutea / harrisoni*, † data from Hume et al. (2015) *Symbiodinium thermophilum sp. nov.*, a thermotolerant symbiotic alga prevalent in corals of the world’s hottest sea, the Persian/Arabian Gulf. *Sci Rep* **5**: 8562

**Supplementary Table S5: Collection site, date and *psbA^ncr^* sequence accession numbers of Gulf C3 coral samples used in the *psbA^ncr^* phylogenetic analysis.**

| **ID** | **Collection Site** | **Collection Date** | **Accession Number** | **Host Species** | **ID** | **Collection Site** | **Collection Date** | **Accession Number** | **Host Species** |
| --- | --- | --- | --- | --- | --- | --- | --- | --- | --- |
| BH1415*1 | Dalma | Sept 2012 | KM458276 | *P. harrisoni* | BH1628 | Umm Al Quwain | March 2013 | KP280255 | *P. lutea* |
| BH1686*1 | Dalma | Sept 2012 | KP280207 | *P. harrisoni* | BH1426*13 | Umm Al Quwain | March 2013 | KM458283 | *P. lutea* |
| BH1448 | Dalma | Sept 2012 | KM458286 | *P. harrisoni* | BH1629*13 | Umm Al Quwain | March 2013 | KP280256 | *P. lutea* |
| BH1413*2 | Dalma | Sept 2012 | KM458274 | *P. harrisoni* | BH1423*14 | Umm Al Quwain | March 2013 | KM458280 | *P. lutea* |
| BH1684*2 | Dalma | Sept 2012 | KP280208 | *P. harrisoni* | BH1630*14 | Umm Al Quwain | March 2013 | KP280257 | *P. lutea* |
| BH1414*3 | Dalma | Sept 2012 | KM458275 | *P. harrisoni* | BH1668 | Umm Al Quwain | March 2013 | KP280258 | *P. lutea* |
| BH1685*3 | Dalma | Sept 2012 | KP280209 | *P. harrisoni* | BH1456*15 | Umm Al Quwain | March 2013 | KM458291 | *P. lutea* |
| BH1450 | Dalma | Sept 2012 | KM458288 | *P. lutea* | BH1626*15 | Umm Al Quwain | March 2013 | KP280259 | *P. lutea* |
| BH1449*4 | Dalma | Sept 2012 | KM458287 | *P. lutea* | BH1669 | Umm Al Quwain | March 2013 | KP280260 | *P. lutea* |
| BH1689*4 | Dalma | Sept 2012 | KP280210 | *P. lutea* | BH1457 | Umm Al Quwain | March 2013 | KP280261 | *P. lutea* |
| BH1692 | Dalma | Sept 2012 | KP280211 | *P. lutea* | BH1660 | Umm Al Quwain | March 2013 | KP280262 | *P. lutea* |
| BH1690 | Dalma | Sept 2012 | KP280212 | *P. harrisoni* | BH1392*16 | Umm Al Quwain | March 2013 | KM458294 | *P. lutea* |
| BH1643 | Dalma | Sept 2012 | KP280213 | *P. harrisoni* | BH1661*16 | Umm Al Quwain | March 2013 | KP280263 | *P. lutea* |
| BH1411 | Dalma | Sep 2012 | KM458273 | *P. harrisoni* | BH1458 | Umm Al Quwain | March 2013 | KM458292 | *P. lutea* |
| BH1647 | Dalma | Sept 2012 | KP280215 | *P. harrisoni* | BH1427*17 | Umm Al Quwain | March 2013 | KM458284 | *P. lutea* |
| BH1691 | Dalma | Sept 2012 | KP280216 | *P. harrisoni* | BH1663*17 | Umm Al Quwain | March 2013 | KP280264 | *P. lutea* |
| BH1447 | Dalma | Sept 2012 | KP280217 | *P. harrisoni* | BH1679 | Umm Al Quwain | March 2013 | KP280265 | *P. lutea* |
| BH1693 | Dalma | Sept 2012 | KP280218 | *P. lutea* | BH1688 | Umm Al Quwain | March 2013 | KP280266 | *P. lutea* |
| BH1416 | Dalma | Sept 2012 | KP280219 | *P. harrisoni* | BH1675 | Ras Al Kaimah | March 2013 | KP280267 | *Porites sp.* |
| BH1702 | Saadiyat | Sept 2012 | KP280220 | *P. lobata* | BH1621 | Ras Al Kaimah | March 2013 | KP280268 | *Porites sp.* |
| BH1704 | Saadiyat | Sept 2012 | KP280221 | *P. lobata* | BH1671 | Ras Al Kaimah | March 2013 | KP280269 | *Porites sp.* |
| BH1708 | Saadiyat | Sept 2012 | KP280222 | *P. lobata* | BH1672 | Ras Al Kaimah | March 2013 | KP280270 | *Porites sp.* |
| BH1356*56 | Saadiyat | Sept 2012 | KM458293 | *P. lutea* | BH1676 | Ras Al Kaimah | March 2013 | KP280271 | *Porites sp.* |
| B359*56 | Saadiyat | Sept 2012 | KP280223 | *P. lutea* | BH1623 | Ras Al Kaimah | March 2013 | KP280272 | *Porites sp.* |
| BH1422*67 | Saadiyat | Sept 2012 | KM458279 | *P. lutea* | BH1620 | Ras Al Kaimah | March 2013 | KP280273 | *Porites sp.* |
| BH1694*67 | Saadiyat | Sept 2012 | KP280224 | *P. lutea* | BH1624 | Ras Al Kaimah | March 2013 | KP280275 | *Porites sp.* |
| BH1451*78 | Saadiyat* | Sept 2012 | KM458289 | *P. lobata* | BH1670 | Ras Al Kaimah | March 2013 | KP280276 | *Porites sp.* |
| BH1695*78 | Saadiyat* | Sept 2012 | KP280225 | *P. lobata* | BH1673 | Ras Al Kaimah | March 2013 | KP280277 | *Porites sp.* |
| BH1417 | Saadiyat | Sept 2012 | KM458277 | *P. lutea* | BH1678 | Ras Al Kaimah | March 2013 | KP280278 | *Porites sp.* |
| BH1419*89 | Saadiyat | Sept 2012 | KM458278 | *P. lutea* | BH1680 | Ras Al Kaimah | March 2013 | KP280279 | *Porites sp.* |
| BH1640*89 | Saadiyat | Sept 2012 | KP280226 | *P. lobata* | BH1677 | Ras Al Kaimah | March 2013 | KP280280 | *Porites sp.* |
| BH1699 | Saadiyat | Sept 2012 | KP280227 | *P. lutea* | BH1683 | Ras Al Kaimah | March 2013 | KP280281 | *Porites sp.* |
| BH1701 | Saadiyat | Sept 2012 | KP280228 | *P. lutea* | BH1497 | Ras Al Kaimah | March 2013 | KP280282 | *Porites sp.* |
| BH1705 | Saadiyat | Sept 2012 | KP280229 | *P. lutea* | BH1625 | Ras Al Kaimah | March 2013 | KP280283 | *Porites sp.* |
| BH1706 | Saadiyat | Sept 2012 | KP280230 | *P. lutea* | BH1682 | Ras Al Kaimah | March 2013 | KP280284 | *Porites sp.* |
| B315 | Saadiyat | Sept 2012 | KP280231 | *P. harrisoni* | BH1681 | Ras Al Kaimah | March 2013 | KP280285 | *Porites sp.* |
| BH1696 | Saadiyat | Sept 2012 | KP280232 | *P. harrisoni* | BH1674 | Ras Al Kaimah | March 2013 | KP280286 | *Porites sp.* |
| BH1641 | Saadiyat | Sept 2012 | KP280233 | *P. lobata* | B21549 | Musandam | March 2013 | KP280287 | *P. lobata* |
| BH1703 | Saadiyat | Sept 2012 | KP280234 | *P. harrisoni* | B21550 | Musandam | March 2013 | KP280288 | *P. lobata* |
| BH1642 | Saadiyat | Sept 2012 | KP280235 | *P. harrisoni* | BH1652 | Musandam | March 2013 | KP280289 | *P. lobata* |
| BH1698 | Saadiyat | Sept 2012 | KP280236 | *P. lutea* | BH1616 | Musandam | March 2013 | KP280290 | *P. lutea* |
| BH1487 | Saadiyat | Sept 2012 | KP280237 | *P. lobata* | BH1617 | Musandam | March 2013 | KP280291 | *P. lobata* |
| BH1418 | Saadiyat | Sept 2012 | KP280238 | *P. lutea* | B21551 | Musandam | March 2013 | KP280292 | *P. lutea* |
| BH1639 | Saadiyat | Sept 2012 | KP280239 | *P. lutea* | B21552 | Musandam | March 2013 | KP280293 | *P. lutea* |
| BH1700 | Saadiyat | Sept 2012 | KP280240 | *P. harrisoni* | B21553 | Musandam | March 2013 | KP280294 | *P. lutea* |
| BH1453*910 | Saadiyat | Sept 2012 | KM458290 | *P. lobata* | B21554 | Musandam | March 2013 | KP280295 | *P. lutea* |
| BH1697*910 | Saadiyat | Sept 2012 | KP280241 | *P. lobata* | BH1649 | Musandam | March 2013 | KP280296 | *P. lobata* |
| BH1636 | Ras Ghanada | Sept 2012 | KP280242 | *P. lutea* | BH1653 | Musandam | March 2013 | KP280297 | *P. lutea* |
| BH1547 | Ras Ghanada | Sept 2012 | KP280243 | *P. lobata* | BH1655 | Musandam | March 2013 | KP280298 | *P. lutea* |
| BH1632 | Ras Ghanada | Sept 2012 | KP280244 | *P. lobata* | B341 | Musandam | March 2013 | KP280299 | *P. lutea* |
| BH1634 | Ras Ghanada | Sept 2012 | KP280245 | *P. harrisoni* | BH1659 | Musandam | March 2013 | KP280300 | *P. lutea* |
| BH1635 | Ras Ghanada | Sept 2012 | KP280246 | *P. harrisoni* | B21556 | Musandam | March 2013 | KP280301 | *P. lobata* |
| BH1548 | Ras Ghanada | Sept 2012 | KP280247 | *P. lutea* | BH1658 | Musandam | March 2013 | KP280302 | *P. lobata* |
| BH1631 | Ras Ghanada | Sept 2012 | KP280248 | *P. harrisoni* | B21557 | Musandam | March 2013 | KP280303 | *P. lobata* |
| BH1633 | Ras Ghanada | Sept 2012 | KP280249 | *P. harrisoni* | BH1656 | Musandam | March 2013 | KP280304 | *P. lobata* |
| BH1664 | Umm Al Quwain | March 2013 | KP280250 | *P. lutea* | BH1614 | Musandam | March 2013 | KP280305 | *P. lobata* |
| BH1425*101 | Umm Al Quwain | March 2013 | KM458282 | *P. lutea* | BH1615 | Musandam | March 2013 | KP280306 | *P. lobata* |
| BH1665*101 | Umm Al Quwain | March 2013 | KP280251 | *P. lutea* | B21561 | Fujairah | Sept 2012 | KP280307 | *P. lobata* |
| BH1662 | Umm Al Quwain | March 2013 | KP280252 | *P. lutea* | BH1550 | Fujairah | Sept 2012 | KP280308 | *P. lobata* |
| BH1428*112 | Umm Al Quwain | March 2013 | KM458285 | *P. lutea* | B21560 | Fujairah | Sept 2012 | KP280309 | *P. lobata* |
| BH1627*112 | Umm Al Quwain | March 2013 | KP280253 | *P. lutea* | B334 | Fujairah | Sept 2012 | KP280310 | *P. lobata* |
| BH1424*1312 | Umm Al Quwain | March 2013 | KM458281 | *P. lutea* | B335 | Muscat | March 2013 | KP280311 | *Porites sp.* |
| BH1666*123 | Umm Al Quwain | March 2013 | KP280254 | *P. lutea* |  |  |  |  |  |

* Asterix-appended IDs with matching numbers refer to sequences obtained from separate PCR reactions from the same coral colony

**Sequences with accession numbers beginning KM are from: Hume et al. (2015) *Symbiodinium thermophilum sp. nov.*, a thermotolerant symbiotic alga prevalent in corals of the world’s hottest sea, the Persian/Arabian Gulf. *Sci Rep* **5**: 8562.

**Supplementary Table S6: *psbA^ncr^* sequence accession numbers and associated ITS2 types used in the *psbA^ncr^* phylogenetic analysis. Sequences were obtained from published studies (*)**.

| **Accession** | **ITS2** | **Accession** | **ITS2** | **Accession** | **ITS2** | **Accession** | **ITS2** | **Accession** | **ITS2** | **Accession** | **ITS2** |
| --- | --- | --- | --- | --- | --- | --- | --- | --- | --- | --- | --- |
| JQ043553 | C26a | JQ043638 | C3 | KF572236 | C7c | KF572293 | C3 | KF572358 | C40 | KF572409 | C3 |
| JQ043555 | C26a | JQ043639 | C3 | KF572241 | C7a | KF572294 | C3 | KF572359 | C40 | KF572410 | C3 |
| JQ043556 | C26a | JQ043640 | C3 | KF572242 | C7a | KF572295 | C3 | KF572360 | C40 | KF572411 | C3 |
| JQ043557 | C26a | JQ043641 | C3 | KF572243 | C7a | KF572296 | C3 | KF572361 | C40 | KF572412 | C3 |
| JQ043558 | C26a | JQ043642 | C3 | KF572244 | C7a | KF572297 | C3 | KF572362 | C40 | KF572413 | C3 |
| JQ043559 | C26a | JQ043643 | C3 | KF572245 | C7a | KF572299 | C3c | KF572363 | C3 | KF572413 | C3 |
| JQ043560 | C26a | JQ043644 | C3 | KF572246 | C7a | KF572300 | C3c | KF572364 | C40 | KF572415 | C87 |
| JQ043561 | C26a | JQ043645 | C3k | KF572247 | C7 | KF572301 | C3 | KF572365 | C40 | KF572416 | C87 |
| JQ043580 | C31c | JQ043646 | C3k | KF572248 | C7 | KF572302 | C3 | KF572366 | C40 | KF572417 | C87 |
| JQ043589 | C26a | JQ043647 | C3k | KF572249 | C7 | KF572303 | C3 | KF572367 | C40 | KF572418 | C87 |
| JQ043591 | C26a | JQ043648 | C3k | KF572250 | C7 | KF572304 | C3 | KF572368 | C40 | KF572419 | C3 |
| JQ043592 | C26a | JQ043649 | C3i | KF572251 | C7 | KF572305 | C3 | KF572369 | C40 | KF572420 | C3 |
| JQ043593 | C26a | JQ043650 | C3 | KF572252 | C7 | KF572306 | C3 | KF572370 | C40 | KF572421 | C3 |
| JQ043594 | C26a | JQ043651 | C3i | KF572253 | C7 | KF572307 | C3 | KF572371 | C31 | KF572422 | C3 |
| JQ043595 | C26a | JQ043652 | C3 | KF572254 | C7 | KF572308 | C3 | KF572372 | C3 | KF572423 | C3 |
| JQ043596 | C26a | JQ043653 | C3 | KF572255 | C7 | KF572309 | C3 | KF572373 | C3h | KF572424 | C3 |
| JQ043598 | C31 | JQ043654 | C3i | KF572256 | C7 | KF572310 | C3 | KF572374 | C3h |  |  |
| JQ043599 | C31 | JQ043655 | C3 | KF572257 | C7 | KF572313 | C3 | KF572375 | C3 |  |  |
| JQ043600 | C31 | JQ043656 | C3 | KF572258 | C7 | KF572314 | C3 | KF572376 | C3 |  |  |
| JQ043601 | C31c | JQ043657 | C3 | KF572259 | C7 | KF572315 | C3 | KF572377 | C3 |  |  |
| JQ043602 | C31 | JQ043658 | C21 | KF572260 | C7 | KF572316 | C3 | KF572379 | C3 |  |  |
| JQ043603 | C31 | JQ043659 | C21 | KF572261 | C7 | KF572317 | C3 | KF572380 | C3s |  |  |
| JQ043604 | C31 | JQ043660 | C21 | KF572262 | C7 | KF572318 | C3 | KF572381 | C3s |  |  |
| JQ043605 | C30 | JQ043661 | C21 | KF572263 | C7 | KF572319 | C3 | KF572382 | C3s |  |  |
| JQ043606 | C31 | JQ043662 | C21a | KF572264 | C7 | KF572320 | C3 | KF572383 | C3s |  |  |
| JQ043607 | C31 | JQ043663 | C21a | KF572265 | C7 | KF572321 | C3 | KF572384 | C3s |  |  |
| JQ043608 | C31 | JQ043664 | C21a | KF572266 | C7 | KF572322 | C3 | KF572385 | C3b |  |  |
| JQ043609 | C31 | JQ043665 | C21a | KF572267 | C7 | KF572323 | C3 | KF572386 | C3b |  |  |
| JQ043610 | C31 | JQ043666 | C21a | KF572268 | C7 | KF572324 | C3 | KF572387 | C3b |  |  |
| JQ043612 | C31 | JQ043668 | C27 | KF572269 | C7 | KF572325 | C3 | KF572388 | C3b |  |  |
| JQ043613 | C31 | JQ043669 | C27 | KF572270 | C7 | KF572326 | C3 | KF572389 | C3b |  |  |
| JQ043615 | C31 | JQ043670 | C27 | KF572271 | C7 | KF572327 | C3 | KF572390 | C3b |  |  |
| JQ043616 | C31 | JQ043671 | C27 | KF572272 | C3 | KF572328 | C3 | KF572391 | C3b |  |  |
| JQ043618 | C17 | JQ043672 | C27 | KF572273 | C3 | KF572329 | C3 | KF572392 | C3b |  |  |
| JQ043619 | C17 | JQ043673 | C27 | KF572274 | C3 | KF572330 | C3 | KF572393 | C3b |  |  |
| JQ043621 | C17a | JQ043674 | C27 | KF572275 | C3 | KF572336 | C3b | KF572394 | C3b |  |  |
| JQ043623 | C17a | KF572222 | C7a | KF572277 | C3 | KF572337 | C3b | KF572395 | C3 |  |  |
| JQ043624 | C21 | KF572223 | C7a | KF572278 | C3 | KF572338 | Cq | KF572396 | C3 |  |  |
| JQ043625 | C21 | KF572224 | C7a | KF572279 | C3 | KF572339 | Cq | KF572397 | C3 |  |  |
| JQ043626 | C21 | KF572225 | C7a | KF572280 | C3 | KF572340 | Cq | KF572398 | C3 |  |  |
| JQ043627 | C3h | KF572226 | C7a | KF572281 | C3 | KF572341 | Cq | KF572399 | C3a |  |  |
| JQ043628 | C3h | KF572227 | C7a | KF572282 | C3 | KF572343 | C3 | KF572400 | C3a |  |  |
| JQ043629 | C3h | KF572228 | C7a | KF572284 | C3 | KF572344 | C3 | KF572401 | C3a |  |  |
| JQ043630 | C3h | KF572229 | C7a | KF572285 | C3 | KF572345 | C3 | KF572402 | C3a |  |  |
| JQ043631 | C3h | KF572230 | C7a | KF572286 | C3 | KF572346 | C3 | KF572403 | C3a |  |  |
| JQ043633 | C21 | KF572231 | C7a | KF572287 | C3 | KF572347 | C3 | KF572404 | C3 |  |  |
| JQ043634 | C3 | KF572232 | C7a | KF572289 | C3 | KF572353 | C3 | KF572405 | C3 |  |  |
| JQ043635 | C3 | KF572233 | C7a | KF572290 | C3 | KF572354 | C3 | KF572406 | C3 |  |  |
| JQ043636 | C3 | KF572234 | C7a | KF572291 | C3 | KF572355 | C3 | KF572407 | C3 |  |  |
| JQ043637 | C3 | KF572235 | C7c | KF572292 | C3 | KF572357 | C40 | KF572408 | C3 |  |  |

(*) LaJeunesse TC, Thornhill DJ (2011). Improved resolution of reef-coral endosymbiont *Symbiodinium* species diversity, ecology, and evolution through *psbA* non-coding region genotyping. *PLoS ONE* **6:** e29013.

Thornhill DJ, Lewis AM, Wham DC, LaJeunesse TC (2014). Host-specialist lineages dominate the adaptive radiation of reef coral endosymbionts. *Evolution* **68:** 352-367.
